# 中国输血依赖型β地中海贫血诊断与治疗指南（2022年版）

**DOI:** 10.3760/cma.j.issn.0253-2727.2022.11.002

**Published:** 2022-11

**Authors:** 中华医学会血液学分会红细胞疾病（贫血）学组

β地中海贫血（β地贫）是由于β珠蛋白基因缺陷导致的遗传性溶血性疾病，是WHO关注的全球社会公共问题之一，我国南方地区如广东、广西、海南、云南、贵州、江西、湖南、四川、重庆、福建、香港、澳门及台湾是β地贫的高发区。20世纪80年代以前输血依赖型β地贫（TDT）被认为是致死性疾病，随着医疗技术及社会经济文化的巨大进步，TDT得到了有效的预防及管理，患者的寿命得到了极大的延长，生存质量得到了显著的提高。为进一步提高我国TDT（特别是成年TDT）的诊断及治疗水平，中华医学会血液学分会征求有关专家的意见，参考最新的国际地中海贫血联盟（TIF）的指南及我国以往的专家共识和相关文献，形成这部指南，旨在进一步规范我国TDT的诊断及治疗。

一、证据水平及推荐等级

本指南参考文献来自PubMed、OVID平台数据库、Springer-Link、Elsevier Science Direct电子期刊、中国期刊网全文数据库（CNKI）和万方数据库等数据资源。文献参照2001年英国牛津循证医学的证据分级与推荐意见强度[1]，将证据水平分为Ⅰ、Ⅱ、Ⅲ、Ⅳ、Ⅴ共5个级别，推荐等级分为A、B、C和D共4个等级。本指南中以［证据水平/推荐等级］表示。

二、TDT病因和病理生理

人类β珠蛋白基因簇定位于第11号染色体短臂1区5带4亚带（11p15.4）。β地贫是由于β珠蛋白基因发生突变，导致β珠蛋白基因的转录、前体mRNA的加工、mRNA的翻译及β珠蛋白肽链的完整性发生障碍，致使β珠蛋白肽链的合成不足或完全不能合成，直接影响正常的成人血红蛋白（Hb A）的合成并引起α珠蛋白肽链与非α珠蛋白肽链的合成比例不平衡，从而导致α珠蛋白肽链相对过剩。相对过剩的α珠蛋白肽链可在红细胞内形成包涵体，导致红细胞膜的氧化损伤，造成红细胞破坏及骨髓的无效造血，临床上引起贫血、黄疸、脾大、骨髓腔扩大引起的地贫外貌等症状及体征，这是β地贫主要病理基础[2]（图1）。

**图1:**
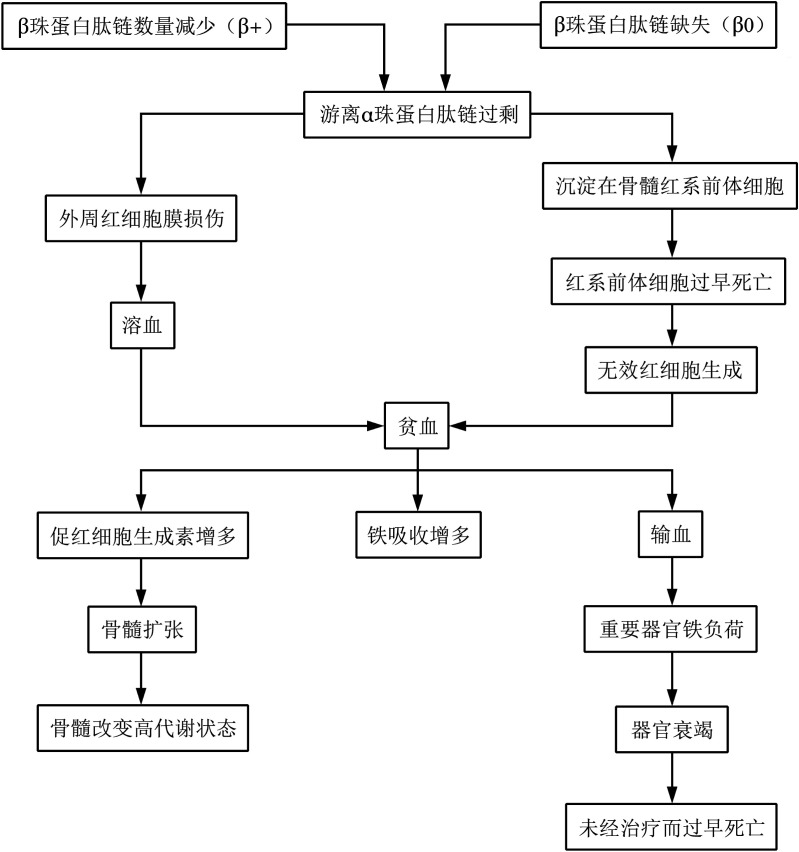
β地中海贫血中游离α珠蛋白肽链过剩产生的影响[2]

正常人自父母双方各遗传一条正常β珠蛋白基因，合成正常量的β珠蛋白肽链。若自父母遗传了异常β珠蛋白基因，则可导致本病。β地贫大部分是点突变，少部分为基因缺失。基因突变致β链的生成完全受抑制者称为β^0^地贫，部分受抑制者称为β^+^地贫[3]。TDT的基因型多为纯合子或复合杂合子状态。染色体上的2个等位基因都有相同致病突变的个体称为纯合子；同源染色体上只有1个致病突变的个体称为杂合子；2个等位基因的致病突变不同的个体称为复合杂合子。若双亲均为β地贫杂合子，其子女获得TDT的概率为25％，杂合子概率为50％，余25％为正常。

三、TDT临床表现

临床根据贫血严重程度和是否需要定期输血将β地贫分为TDT和非输血依赖型β地贫（NTDT）。TDT患者需要定期输血才能存活，如果没有足够的输血支持会出现一系列并发症，并且生存期很短。TDT患者包括重型β地贫、少数中间型β地贫、严重HbE/β地贫。NTDT患者包括轻型及中间型β地贫、HbE/β地贫。轻型患者一般无症状或只有轻度贫血，多在家系调查时被筛查发现。中间型多于幼儿期出现中度贫血，但严重度不及重型，少数中间型β地贫可进展为TDT。重型β地贫又称Cooley贫血，出生后3～6个月开始出现临床症状，且呈慢性进行性加重，早期症状为食欲不佳、喂养困难、腹泻、易激惹、发育缓慢、体重不增；随后面色逐渐苍白，肝脾特别是脾脏进行性肿大，腹部逐渐膨大；1岁后体征越来越明显，贫血进行性加重，巩膜黄染、生长发育迟缓、身材矮小、肌肉无力、骨骼变形、头颅增大，额部、顶部、枕部隆起，颧骨隆起，鼻梁塌陷，上颌及牙齿前突，形成典型的“地贫外貌”。巨脾可继发脾功能亢进而引起血细胞减少，时常有感染、发热、鼻出血等。症状体征随年龄增长而日益明显。TDT患者长期反复输血可致继发性铁过载，过多的铁沉着于心肌、肝、胰腺和脑垂体等，引起器官功能受损并出现相应症状，包括合并凝血功能障碍、糖代谢异常、生长发育迟缓、骨质疏松等；其中最严重的是心力衰竭，是导致地贫患者死亡的主要原因之一。

近20年来，经推广规范的输血和祛铁治疗，大多数TDT患者不再出现本病典型特征的临床症状和体征，生存期明显延长，逐渐进入成年期。

四、TDT诊断

1. 临床表现：首诊时典型的临床特征。

2. 血液学改变：①外周血HGB<70 g/L，呈小细胞低色素性贫血，红细胞平均容积（MCV）<80 fl、红细胞平均血红蛋白（MCH）<28 pg、红细胞平均血红蛋白浓度（MCHC）<320 g/L。红细胞形态不一、大小不等、中央淡染区扩大，出现靶形红细胞和红细胞碎片，嗜碱性点彩红细胞、多嗜性红细胞、有核红细胞增加，网织红细胞比例增高。部分患儿由于骨髓造血功能代偿增加可致PLT增高。脾功能亢进时，WBC和PLT减低。②骨髓象呈红细胞系统增生明显活跃，以中、晚幼红细胞占多数，成熟红细胞改变与外周血相同。③红细胞渗透脆性明显降低。

3. 血红蛋白分析：未启动治疗（输血）前，血红蛋白电泳显示胎儿血红蛋白（Hb F）显著增高，一般达30％～90％，是诊断TDT的重要依据。部分患者血红蛋白A2（Hb A2）含量升高。Hb F不增高应排除近期输血的影响，可在输血后复查。

4. 区域及家系调查：区域调查显示患者来自地贫高发区域。患者父母亲外周血常规呈小细胞低素性贫血，血红蛋白电泳示Hb A2含量升高（3.5％～6.0％），Hb F多正常；基因检测证实为β地贫基因携带者。

5. 基因诊断：可采用等位基因特异性寡核苷酸探针点杂交（PCR-ASO）、反向点杂交（RDB）和DNA测序等方法检测β地贫基因缺陷的类型。目前世界范围内已发现300多种β珠蛋白基因突变类型，中国人群中已发现50多种，目前国内实验室多应用反向点杂交方法进行地贫的基因诊断，但该方法主要针对我国常见的珠蛋白基因突变类型进行检测。因此，临床诊断成立的TDT，且基因不是已知突变类型者，应在明确家族调查资料证实父母均为轻型β地贫的背景下，开展基因测序。

6. 诊断与鉴别诊断：典型的TDT患者诊断并不困难。对于进行性严重贫血的患儿，有脾脏肿大，MCV、MCH、MCHC明显降低，网织红细胞比例增高，外周血涂片显示红细胞大小不均、有靶形红细胞，红细胞渗透脆性降低，Hb F含量显著增高，大多可以确诊。家族史和籍贯对诊断有重要意义，必要时作颅骨X线检查及血红蛋白分析，疑似病例需作基因诊断（图2、3）。需与缺铁性贫血、巨幼细胞性贫血、新生儿黄疸、红细胞葡萄糖-6-磷酸脱氢酶（G-6-PD）缺乏症、遗传性球形红细胞增多症、再生障碍性贫血、幼年型粒-单核细胞白血病等相鉴别。

**图2:**
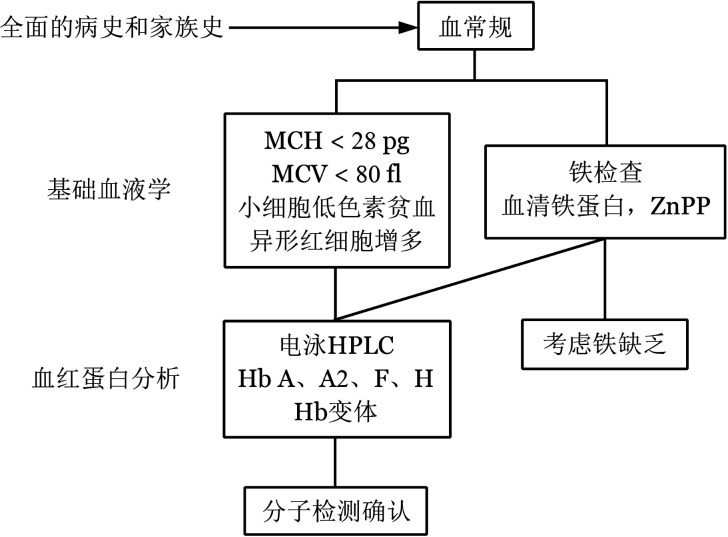
地中海贫血诊断流程图[2] 注　MCH：红细胞平均血红蛋白；MCV：红细胞平均体积；HPLC：高效液相色谱；ZnPP：锌原卟啉

**图3:**
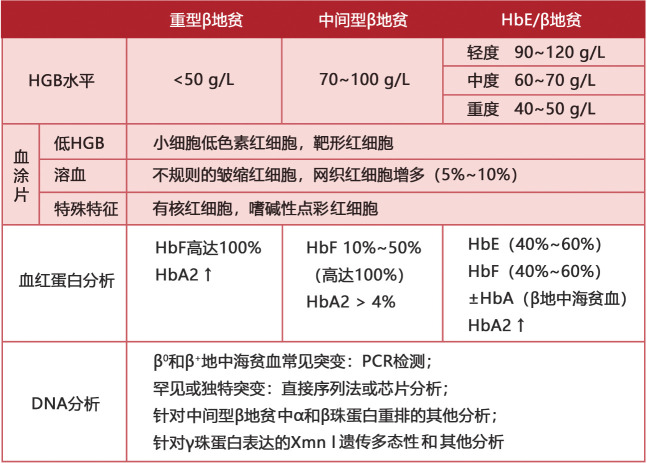
地中海贫血的诊断方法总结（HbE/β地贫：血红蛋白E/β地贫）[2]

五、TDT治疗

规范性输血和去铁治疗是维持TDT患者生存的主要方法，造血干细胞移植（HSCT）是目前临床根治的唯一方法，基因治疗及新药是地贫治疗领域发展的新手段。

（一）输血疗法

目的在于维持患者血红蛋白浓度接近正常水平，减轻代偿性骨髓增生及髓外造血，减少肠道铁吸收，防止慢性缺氧，保障患者正常生长发育及改善生存质量的需求。

1. 输血计划：初次输血指征：（1）明确地贫诊断。（2）存在如下情况：①明显的贫血症状；②生长发育缓慢/生长受限；③过多髓内造血产生的并发症，如病理性骨折和面部改变；④具有临床意义的髓外造血［Ⅴ/D］。

输血频率及目标值：（1）每2～5周输血1次，每次输红细胞0.5～1.0单位/10 kg（国内将200 ml全血中提取的红细胞定义为1单位），输血时间根据输血反应和心功能状态调整，宜4 h内输完，但可依据实际情况适当延长；（2）维持输血前HGB水平在95～105 g/L（通常要使输血后HGB达到130～150 g/L），这可保障正常生长发育及体育活动[2]；（3）维持输血前HGB在110～120 g/L，更适合于心脏病、出现临床显著髓外造血或其他疾病，以及骨髓增生未得到充分抑制的患者；（4）重度贫血患者，每次输注红细胞量宜少，速度宜慢，可少量多次［Ⅴ/D］。

2. 血液制品的选择［Ⅱ/B］[2]：（1）选择ABO及Rh（D）血型相同的红细胞制品，有条件时还可选择与抗原C、E及Kell（Mur）相匹配的红细胞制品；（2）推荐使用去白细胞悬浮红细胞；（3）对合并自身免疫性溶血性贫血者应选择洗涤红细胞；（4）避免应用血缘相关亲属的血液。

（二）祛铁治疗

1. 铁过载评估：TDT患者因长期反复输血，以及长期贫血，肠道铁吸收过多导致继发性铁过载，是地贫常见并发症。目前评估铁过载状况的方法主要有：血清铁蛋白（SF）、肝铁浓度（LIC）及心铁浓度（MIC）等。

SF检测是反映机体铁过载状况最简单实用的方法，SF升高提示铁负荷增加，但需排除感染、肝功能损害、肿瘤、溶血、酗酒等因素影响。建议每3～6个月动态检测SF 1次［Ⅴ/D］。

近年，MRI已广泛应用于评估地贫患者体内脏器铁过载情况。肝脏MRI T2*可反映肝脏铁负荷情况，并与LIC检测存在相关性：LIC 3～7 mg Fe/g干重提示轻度铁过载；7～15 mg Fe/g干重提示中度铁过载；>15 mg Fe/g干重提示重度铁过载［Ⅰ/A］。LIC需每1～2年评估1次［Ⅴ/D］。MIC目前常用心脏MRI T2*值评估：T2*<10 ms提示患者心脏有重度铁过载，建议每3个月复查1次；T2*值位于10～20 ms，提示患者心脏有轻至中度铁过载，建议每年复查1次；T2*>20 ms提示患者心脏暂无明显铁过载，可每2年复查1次[4]［Ⅱ/B］。

肝穿刺活检后通过原子吸收光谱学测定LIC是评价机体铁负荷状况的金标准，该检测方法的敏感性及特异性均高，同时还可进行肝组织病理学分析，判断有无肝纤维化等。但肝穿刺活检为创伤性检查、操作复杂，有出血倾向的患者不适用。

2. 祛铁治疗的时机：（1）输血次数≥10次；（2）在排除活动性炎症、肝病、肿瘤、溶血、酗酒等因素影响后，SF >1000 µg/L或LIC ≥5 mg Fe/g干重。祛铁治疗后每3～6个月监测SF或MRI，当SF <500 µg/L或LIC <5 mg Fe/g干重可暂停使用铁螯合剂[2]［Ⅴ/D］。

在3岁前开展祛铁治疗，要特别监测生长和骨骼发育情况，并且减少剂量。

3. 祛铁治疗策略：

祛铁药物及其选择：目前临床上应用的铁螯合剂主要包括去铁胺（DFO）、去铁酮（DFP）和地拉罗司（DFX）。

（1）DFO：DFO代谢半衰期为20～30 min，代谢后主要通过尿液排出。DFO为大分子物质，肠道无法吸收。

用药方法[2]：①将DFO配成10％的浓度，推荐采用输液泵持续皮下注射，每次输注时间8～12 h［Ⅰ/A］。②儿童标准剂量为20～40 mg·kg^−1^·d^−1^，成人剂量可高达50～60 mg·kg^−1^·d^−1^，每周连续应用5～7 d［Ⅰ/A］。③维生素C与DFO联合应用可增强其从尿中排铁的作用，维生素C剂量2～3 mg·kg^−1^·d^−1^［Ⅱ/B］。④如SF持续升高>2500 µg/L或LIC>15 mg Fe/g干重或心脏MRI T2*值<10 ms，或出现明显铁相关性心肌病（左室射血分数<55％，心律失常或心力衰竭）或骨髓移植前的患者，需强化或挽救祛铁治疗（具体见“祛铁治疗方案”）［Ⅱ/B］。

治疗指数（毒性指数）＝平均每日DFO剂量（mg/kg）/血清铁蛋白（µg/L）。应保持治疗指数<0.025，以减少DFO的不良反应［Ⅰ/A］。建议每年使用DFO至少225 d[2]［Ⅱ/B］。

注意事项及不良反应：①用药前后应监测血清铁蛋白。②皮下注射部位首选腹部，每天应更换腹部注射部位，以助药物吸收。③维生素C可动员铁及氧化代谢并间接影响心肌细胞，在重度铁过载时不宜使用大剂量维生素C；停用DFO期间也应停止服用维生素C［Ⅱ/B］。④DFO偶见过敏反应，长期使用偶可致白内障和长骨发育障碍，剂量过大可引起视力和听觉减退。建议检查生长发育及骨发育，定期检测视力及听力［Ⅴ/D］。⑤对DFO依从性不好或不耐受的患者，可换用DFP或DFX。

（2）DFP：DFP是口服铁鳌合剂，代谢半衰期为3～4 h，主要经尿液排出。研究表明DFP对心脏铁过载有较强的治疗作用[5]［Ⅰ/A］。

用药方法：①标准剂量为75 mg·kg^−1^·d^−1^，分3次口服，最大剂量不超过100 mg·kg^−1^·d^−1^[2]［Ⅰ/A］。②适用于6岁以上的患儿。

注意事项及不良反应[6]：①目前维生素C在DFP治疗中的联合作用尚未明确，不推荐联合应用。②DFP常见的不良反应是关节痛（主要是大关节）、一过性的丙氨酸转氨酶升高、胃肠道反应和锌缺乏。③严重不良反应为粒细胞减少（ANC<1.5×10^9^/L）和粒细胞缺乏（ANC<0.5×10^9^/L），建议定期监测外周血常规［Ⅴ/D］。④DFP会引起听力及视力受损，建议定期监测听力，必要时每年进行1次眼科检查，包括视网膜评估。⑤DFP可透过血脑屏障，过量会引起神经系统损伤（>230 mg·kg^−1^·d^−1^），但较为罕见。

（3）DFX：DFX为口服铁螯合剂，代谢半衰期8～16 h，主要经粪便排出。用药方法：①DFX常用剂量为20～40 mg·kg^−1^·d^−1^。②适用于2岁以上的患儿，每日1次，餐前口服。

注意事项及不良反应[7]：①DFX可引起胃肠道反应、皮疹、丙氨酸转氨酶升高，偶有听觉减退。②DFX还可引起肌酐升高，建议定期检查肾功能，肾功能不全时应慎用，若尿蛋白/肌酐比值明显上升，超过1 mg/g，应考虑中断或减量［Ⅴ/D］。

（4）联合用药：单独应用铁螯合剂而祛铁疗效不佳，可予2种铁螯合剂联合应用。联合策略包括应用DFO和DFP、DFP和DFX、DFO和DFX[5],[8]。TDT合并急性心力衰竭者建议在高剂量连续静脉滴注DFO基础上口服DFP或DFX治疗[5],[8]［Ⅱ/B］。

（5）祛铁治疗方案见图4。

**图4:**
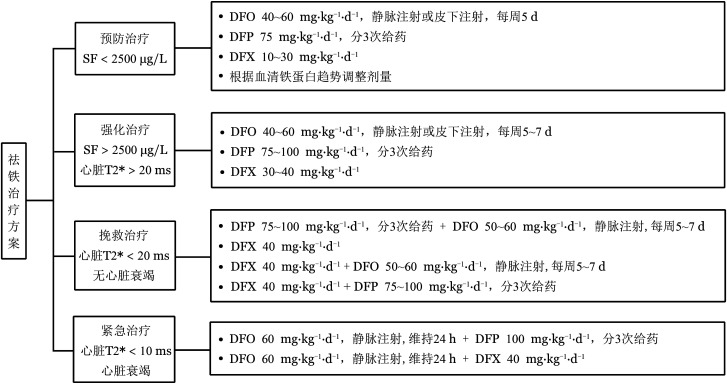
地中海贫血患者祛铁治疗方案 注 SF：血清铁蛋白；DFO：去铁胺；DFP：去铁酮；DFX：地拉罗司

（三）改善无效造血

罗特西普（luspatercept）是一种晚期红细胞成熟剂，可促进β地贫患者骨髓内幼红细胞向晚期红细胞分化成熟，适用于治疗需要定期输注红细胞且红细胞输注≤15单位/24周的≥18岁β地贫成人患者（境外临床研究中1单位红细胞指200～350 ml浓缩红细胞，应根据中国的临床实践进行换算）；推荐起始剂量为1.0 mg/kg，皮下注射，每3周1次；以1.0 mg/kg起始剂量至少连续给药2次（6周）后未达到红细胞输注负荷降低，则应将剂量增加至1.25 mg/kg。最大治疗剂量不应超过每3周1.25 mg/kg［Ⅰ/A］。罗特西普常见不良事件包括一过性骨痛、关节痛、眩晕、高血压和高尿酸血症，大多数为轻中度[9]［Ⅰ/A］。目前，罗特西普对儿童TDT患者的疗效和安全性的相关研究正在进行中。

（四）HSCT

HSCT是目前根治TDT的唯一方法。我国主要地贫移植中心的地贫移植无地贫生存（TFS）率达90％以上，是TDT治疗的最佳选择。根据供者来源、人类白细胞抗原（HLA）不同，地贫移植分为HLA全相合同胞供者移植（MSD-HSCT）、非血缘供者移植（UD-HSCT）和HLA单倍体移植（Haplo-HSCT）；根据干细胞来源分为骨髓移植（BMT）、外周血干细胞移植（PBSCT）和脐带血移植（UCBT）。

1. 移植前危险度评估及移植时机[10]：既往国际多采用Pesaro评分，根据有无肝肿大（肋缘下≤ 2 cm）、肝纤维化和铁螯合剂是否规则应用分成三个危险度。我国重型地贫患者绝大多数属于Ⅱ度及以上，少有Ⅰ度。因肝活检为侵入性操作，故Pesaro分级方案在我国的临床应用有一定的局限性。因此移植前规则输血及规则祛铁治疗尤为关键。HSCT最佳年龄为2～7岁，年龄≥16岁是移植的高危因素［Ⅰ/A］；此外，患者年龄≥7岁同时伴肝右肋缘下≥5 cm也是移植的高危因素,移植相关死亡率增加。对有HLA全相合供者、年龄16岁以下、器官功能正常、没有严重感染的TDT患者，宜尽快考虑移植。

2. 移植供者及干细胞选择[11]：（1）人类白细胞抗原（HLA）配型选择供者：选择顺序是HLA全相合同胞供者➝非血缘HLA全相合供者➝单倍体供者。有经验的HSCT中心可考虑进行非血缘或单倍体HSCT。HLA全相合同胞BMT和PBSCT植入率高，而UCBT则应保证一定阈值的单个核细胞数（MNC）和CD34^+^细胞数［Ⅱ/B］。（2）不同移植方式的MNC和CD34^+^细胞阈值推荐：BMT时要求MNC为（2～4）×10^8^/kg，CD34^+^细胞为（2～4）×10^6^/kg；PBSCT时MNC≥ 4×10^8^/kg，CD34^+^细胞≥ 4×10^6^/kg；UBCT时MNC≥ 3.7×10^7^/kg，CD34^+^细胞≥ 2.3×10^5^/kg，方才有利于植入［Ⅴ/D］。

3. 移植预处理方案[12]：经典清髓方案为白消安（BU）及环磷酰胺（Cy）。为减少移植物排斥及移植物抗宿主病（GVHD），预处理方案中可酌情加用抗胸腺细胞球蛋白（ATG）、氟达拉滨（Flu）和塞替派（TT）［Ⅲ/B］。国内常用的移植预处理方案包括：广西医科大学第一附属医院的BU+Cy+Flu+ATG方案（GX-07-TM）[13]和南方医科大学南方医院的BU+Cy+Flu+TT方案（NF-08-TM）[14]等。

4. GVHD的预防方案：HLA全相合同胞供者移植建议采用环孢素（CsA）+短程甲氨蝶呤（MTX）+霉酚酸酯（MMF）方案[13]；术后维持CsA血药浓度（200±50）mg/L，如无GVHD表现，移植术后12个月起缓慢减量，至术后18个月停用，取得很好的临床效果，无关供者移植和单倍体移植建议选择他克莫司（FK506）+MTX+MMF方案[15]或选择PTCY方案[16]［Ⅲ/B］。FK506的目标谷浓度范围为（10±5）µg/L。

5. 肝静脉闭塞病（VOD）的防治方案：地贫患者因肝铁过载等原因，VOD发生率高达10％[17]，应十分注意VOD的预防、早期诊断及治疗。VOD预防可采用肝素、前列地尔、熊去氧胆酸等；治疗方面，除可应用肝素、前列地尔和熊去氧胆酸治疗外，应及早暂停环孢素或他克莫司，有利于防止VOD病情进展恶化，改用甲泼尼龙联合CD25单抗等加强GVHD预防，并停用损害肝功能的药物。待病情缓解后再尝试改用环孢素或他克莫司或改用其他免疫抑制剂[17]。

6. 移植后嵌合状态：移植后监测供者植入百分比对预测移植排斥或移植失败有重要的临床意义。移植后嵌合状态分3个水平：嵌合程度1（受者细胞百分比<10％），嵌合程度2（受者细胞百分比占10％～25％），嵌合程度3（受者细胞百分比>25％）。宿主残余造血干细胞比例升高会增加移植物排斥风险，当出现嵌合程度3时，患者存在较高移植物排斥风险。对嵌合程度1患者，应严密观察；对嵌合程度2患者，可减停免疫抑制药物；对嵌合程度3患者，可应用供者淋巴细胞或供者干细胞输注，建议造血干细胞植入后3个月内每周监测嵌合状态，以后可每1～3个月进行监测，直到移植后2年[18]［Ⅱ/B］。

（五）脾切除

脾切除术为治疗TDT患者的姑息手段，脾切除的指征为[2]：（1）依赖输血量明显增多，如维持HGB 90～105 g/L，每年红细胞输注量>200 ml/kg者，且经规则祛铁治疗而铁负荷仍增加［Ⅱ/B］。（2）脾功能亢进者，患者出现红细胞破坏增加，持续的白细胞减少或血小板减少，临床上出现反复感染或出血。（3）脾脏增大并有伴随症状者，如患者出现明显左上腹疼痛或易饱感，巨脾引起压迫及脾破裂等可能。符合以上指征之一可行脾切除术，建议行脾切除术时患者年龄≥5岁，5岁以下进行脾切除会增加严重脓毒症发生风险。

全脾切除的外科手术有开腹和腹腔镜两种方法。腹腔镜手术显著降低了术后30 d的死亡率，缩短住院时间，显著减少肺部、伤口感染并发症。脾切除后暴发性感染是手术的严重并发症，需加强抗感染治疗。术前和术后使用广谱抗生素。建议术前4～6周或至少提前2周完成免疫接种，包括肺炎链球菌、B型流感嗜血杆菌、脑膜炎奈瑟菌等疫苗，并在术后重复接种（B型流感嗜血杆菌尚未推荐重复接种）。建议术后每年接种流感病毒疫苗［Ⅱ/B］。血栓形成也是脾切除术后的常见并发症，建议定期监测凝血功能及血常规，处于高凝状态或血小板明显增高的患者可予抗凝剂治疗[2]［Ⅱ/B］。肺动脉高压在TDT中也越来越常见，年龄增长和脾切除术是主要危险因素。在许多患者中，脾脏铁负荷接近肝脏铁负荷，输血依赖型地贫患者脾脏切除后，铁会重新定向并积聚在肝脏、心脏和其他器官中，除非使用有效祛铁治疗方案，这些器官的铁负荷将会增加，建议地贫患者脾脏切除后应重新评估脏器铁负荷情况[2]。

（六）基因治疗

地贫是单基因遗传性疾病，是基因治疗的理想对象。目前β地贫的基因治疗可分为基因替代疗法和基因编辑疗法两种。基因替代疗法是采用慢病毒载体将正确的β珠蛋白基因导入患者造血干细胞，并回输给患者，达到提升β珠蛋白表达，促使Hb A生成的目标。此类代表性药物是美国蓝鸟生物公司的“LentiGlobin”（Zynteglo），于2019年在欧洲有条件批准上市。基因编辑疗法是通过抑制γ珠蛋白基因簇的调控因子BCL11A从而提高γ珠蛋白基因表达。通过CRISPR基因编辑BCL11A，以提升γ珠蛋白基因表达，改善患者贫血状况。瑞士CRISPR Therapeutics公司于2018年8月31日启动了全球首个基于CRISPR基因编辑技术提高γ珠蛋白表达的临床试验，受试患者平均随访8.7个月，绝大部分患者的Hb F表达显著提高，部分患者的总HGB量大于100 g/L[19]。国内多家医疗机构先后利用CRISPR-Cas9技术靶向γ珠蛋白编辑靶点，进行了基因编辑治疗TDT患者的临床研究，均已取得初步成效，共完成10余例TDT基因治疗，患者Hb F在移植后1个月开始出现显著上升，使部分TDT患者摆脱输血依赖，研究随访仍在进行中。然而，目前基因治疗临床经验有限，需要更多的临床数据和大规模试验来证明基因治疗是一种安全和治愈性的治疗方法。

（七）探索性治疗

近10年来，随着对沙利度胺治疗β地贫机制研究的深入，以及临床试验的不断更新，沙利度胺被证实是一种有前景的Hb F诱导剂，可改善β地贫患者的血红蛋白水平及减少输血依赖，其疗效尚未得到广泛共识[20]［Ⅱ/B］。目前沙利度胺仅在成人患者中有使用经验，使用剂量为50～100 mg/d［Ⅴ/D］。沙利度胺治疗β地贫的有效性及安全性仍处于不断探索阶段，包括最佳有效剂量、是否改善器官铁过载、作用机制等问题尚未得到解决，仍需要更多大样本的随机对照临床研究证实。

六、TDT的预防

TDT危害大，临床治疗成本高，效果仍不理想。因此，预防控制显得尤为重要。预防控制的主要措施包括社区筛查、遗传咨询和产前诊断。对于有TDT患儿出生史、夫妻均为地贫携带者的高危孕妇应严格进行产前诊断。产前诊断包括取胎儿绒毛、羊水及胎儿脐带血作基因分析。其中以早期绒毛为首选，取胎儿绒毛以孕8～12周为最佳时间；若错过采集绒毛的时机，可于孕16～24周采集羊水，并经培养去除母血细胞后提取胎儿DNA进行基因分析；经胎儿脐静脉穿刺取血样提取胎儿DNA也可以用于产前诊断，一般在孕20～26周进行。但上述均为侵入性产前诊断途径，存在一定风险。近年来逐渐发展了一种非损伤性、非侵入性产前诊断方法，即从孕妇外周血中提取胎儿DNA用于基因分析。产前诊断是预防与监控的结果，具有很强的社会意义和实用价值，特别是在地贫高发区，是预防地贫的关键。
